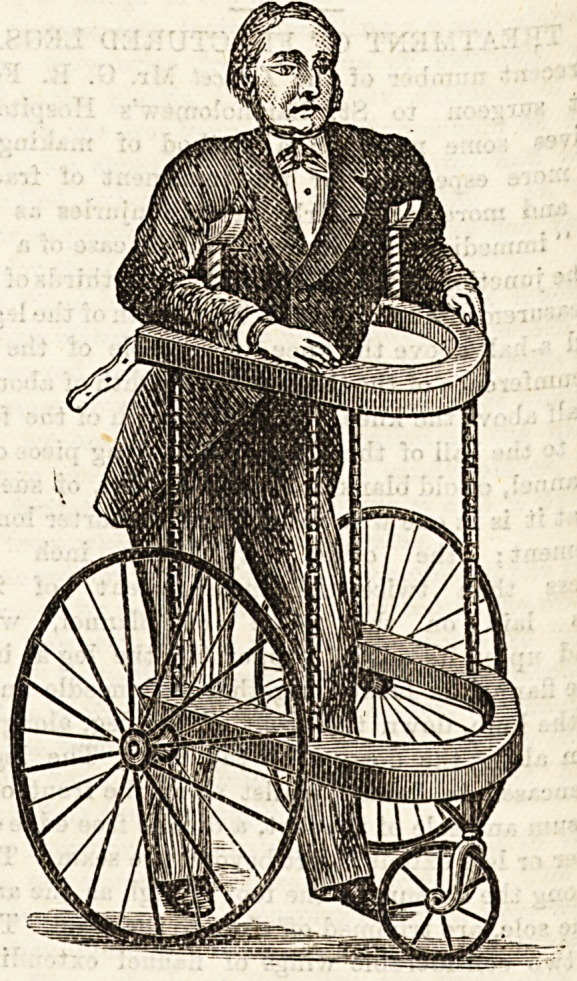# New Drugs, Appliances, and Things Medical

**Published:** 1890-08-16

**Authors:** 


### NEW DRUGS, APPLIANCES, AND THINGS MEDICAL.

[All preparations, appliances, novelties, etc., of which a notice is desired, should be sent for The Editor, to care of The Manager, 140, Strand, London, W.C.]

### "FLORADOR."

We have received samples of the above from the Florador Food Company, Southampton Street, Holborn, for notice. Having tested them both microscopically and chemically, we are prepared to give a very favourable opinion of this wheat food. It is made in three different sized grains—large, medium, and fine—which can be used for the different classes of comestibles in which wheat products play an important part. We think also that, as it is a wheat food properly prepared, the finest grain will do well for infants' food where the carbohydrate element, combined with a good proportion of nitrogenous material, is required. Having satisfied ourselves as to the purity of this production we asked our cook to try the remainder of the samples, so as to give us an idea of the food when prepared for the table. Some excellent fritters and cheese souffle were the result of the trial, and we can distinctly state that we never tasted better. Our domestic informed us that "Florador" was easy to work with, and well repaid the care expended on its combinations by their appetising appearance, and by the appreciation with which they were received. We note that at the Cookery Competition at the Westminster Town Hall last week Florador received the only gold medal for this class of food stuffs.

### ALFRED CARTER'S INVALID FURNITURE.

At the request of Mr. Carter, we recently paid a visit to his show rooms, 47, Holborn Viaduct. We were first shown a number of the smaller appliances which go to make the life of the invalid or convalescent more tolerable and pleasant. Among them was Barraclough's portable bed-table. This is a most admirable invention. It consists of an ordinary bed-table, the legs of which are telescopic, but in addition there is a mirror with candles. By moving the glass a view of everything on the table, or in its vicinity is obtained. Though reversed in position the patient soon learns to allow for this and correctly estimates its relative situation. We need hardly state what an enlarged horizon of view this gives the unfortunate child or adult who is compelled to pass many weary weeks on his back. For children, especially, this will be found valuable, for by its means the little patients can play with their toys and also see to a considerable extent what is going on in the room around them. Mr. Carter also makes a very convenient self-balancing lamp for fixing to this or other forms of bed-table. The same kind of lamp, not balancing, is supplied with a firm stand, on the rod of which it slides up and down, which makes it very suitable for ordinary reading or for the consulting-room. Both lamps have an ingenious reflector attached.

We were then shown all manner of invalid bed-room requisites, till we thought that with such helps as these, it was almost impossible to want anything when laid up There were reading machines and desks in infinite variety, bed cradles to keep off the weight of the clothes, or to keep the patient cool, a bed-lift to enable the clothes to be changed and the patient sponged and dried, chairs to move the patient in when he was getting better, or if he had chronic heart disease, with dropsy, there was a marvellous movable chair with a leg rest of most simple construction, in which he could sleep and be as comfortable as his distressing complaint would allow him to be.

Then we came to a big room full of couches and mechanical chairs. Some of these, from the engineering devices shown in their construction, looked something like the dreaded rack of old, but on trying them it was evident that, far from producing discomfort and pain, they tended to render the life of those already suffering much easier and more tolerable. Again, these couches and chairs are not of the Procrustean type, for they can be adapted to the stature of their users. We were particularly struck with an invalid's couch, with screw and rack movements combined; a new combination couch which can be easily made into an armchair, and can be packed into a small compass for travelling, a great desideratum for the invalid, and also with an admirable hospital carrying-chair. We would draw the attention of the profession to a very complete gynæcological and consulting-room chair. Mr. Carter, has succeeded, we think, in making one that fulfils everything that is required in this direction.

The next thing we were shown was a "walking machine" for the semi-paralyzed. It is a very clever adaptation of the old "go cart" of our grandparents' time, put on to wheels, with crutches fitting under the arms of the patient. By it he can get about in an erect position, thus giving variety to the limited movements of these unfortunates. We give an illustration, and think it likely to add to pleasures of the somewhat dreary lives of those who otherwise would be compelled to go in a chair or be lifted about. Of chairs Merlin, or otherwise, Mr. Carter showed us an endless variety adapted to the means of all classes of society. There was a specially strong useful one fitted for hospital or infirmary work, which took our fancy very much. Then there were commode chairs of all kinds, bed-rests, bed-lifts, and the thousand-and-one necessaries of the sick-room, in fact, such a collection or armamentarium that one wonders what our ancestors did without them, for here are to be seen all the mechanical aids and appliances which we call into use whenever laid up by fractures, paralyses, or long sicknesses, and without which no well-appointed sick-room "is considered complete." Finally, Mr. Alfred Carter took us to have a look at his ambulance appliances. These are numerous and of all sorts; chief amongst them is his Viaduct Ambulance Litter, by which the patient can be conveyed on road, rail, or steamer. This has been so highly thought of that the "Hospitals Association Ambulance," which is connected with the County Council, the police, and chief railways, has ordered Mr. Carter to supply them entirely with it. Higher praise cannot be given. We were greatly pleased with the contrivance; it is at once light and safe, and requires no long printed list of directions. By it a patient can be lifted off his bed on to its stretcher, put on the carriage, wheeled to the station, and then directly into a guard's or luggage van, and so conveyed to his destination without shifting from one couch to another, and also without jarring, as the springs are well adjusted. In conclusion, we advise anyone wanting invalid appliances to go to the Viaduct Stores and have a look round; they will meet with every attention, and a keenly-trained mechanical intellect, eager to fix upon any point likely to aid suffering humanity.

THE "DORIS" SAFETY BELT FOR CHILDREN.

This belt has been invented by Mrs. H. M. Peak, of 7, Wesley Street, Liverpool, for the safety of young children. It can be used as a support when teaching a child to walk, or in the perambulator, where it gives the most restless infant great freedom of action for its limbs, though it prevents it from falling out. Mothers and nurses who have tried it speak very highly of the "Doris" belt. They find it also useful if habitually worn by the child, who may be secured by the webbing to the rail of a cot, thus leaving plenty of room to move about, whilst it prevents any risk of over-balancing, and so secures a restless child safely in its bed. The two great recommendations of the "Doris" belt are free play for the limbs and perfect safety for the child.

## Figures and Tables

**Figure f1:**
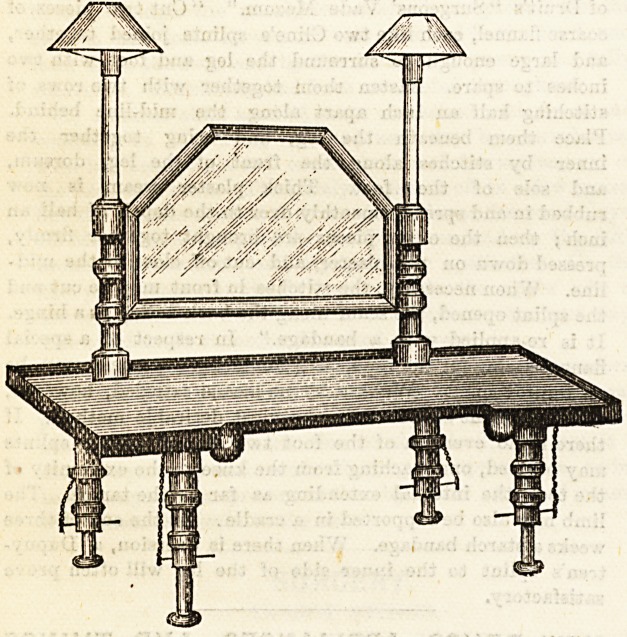


**Figure f2:**